# $CO_2$ footprint minimization of solar-powered HALE using MDO and eco-material selection

**DOI:** 10.1038/s41598-023-39221-3

**Published:** 2023-07-25

**Authors:** Edouard Duriez, Víctor Manuel Guadaño Martín, Joseph Morlier

**Affiliations:** 1 ICA, Université de Toulouse, ISAE-SUPAERO, MINES ALBI, UPS, INSA, CNRS, 3 Rue Caroline Aigle, 31400 Toulouse, France; 2 Université de Toulouse, ISAE-SUPAERO, 10 avenue edouard Belin, 31400 Toulouse, France

**Keywords:** Aerospace engineering, Mechanical engineering

## Abstract

Multidisciplinary Design Optimization (MDO) enables one to reach a better solution than by optimizing each discipline independently. In particular, the optimal structure of a drone varies depending on the selected material. The $CO_2$ footprint of a solar-powered High Altitude Long Endurance (HALE) drone is optimized here, where the structural materials used is one of the design variables. Optimization is performed using a modified version of OpenAeroStruct, a framework based on OpenMDAO. Our EcoHale framework is validated on a classical HALE testcase in the MDO community (FBhale) constructed using high-fidelity codes compared to our low-fidelity approach. The originality of our work is to include two specific disciplines (energy and environment) to adapt to a new problem of $CO_2$ minimization. The choice of eco-materials is performed in the global MDO loop from a choice of discrete materials . This is achieved through a variable relaxation, enabling the use of continuous optimization algorithms inspired from multimaterial topology optimization. Our results show that, in our specific case of electric drone, the optimal material in terms of $CO_2$ footprint is also the optimal material in terms of weight. It opens the door to new researches on digital microarchitectured materials that will decrease the $CO_2$ footprint of the drone.

## Introduction

High Altitude Long Endurance (HALE) drones driven by solar power could be an alternative to satellites for some missions. Their solar power cells and batteries enable them to fly for a few years, which added to their high altitude (above 20 km) make them fit for missions similar to those of satellites^1^. HALE drones can offer permanent coverage of a point or be re-positioned, and are repairable, unlike satellites, which are in orbit. Their lower altitude can offer better resolution for earth observation, but also results in smaller coverage. Their biggest advantage is their lower cost compared to satellites. HALE drones could also be more environmentally friendly as they do not require a high energy consuming launcher. This advantage can be enhanced if a special attention is paid to their environmental impact. For a fully electric HALE drone, most of this impact comes from the materials used and the manufacturing of the drone. Multidisciplinary Design Analysis and Optimization (MDAO) often compressed to MDO enables a better solution to be reached than by optimizing each discipline independently by developing efficient architecture^2^. The most advanced numerical framework is developed by NASA^3^. MDO has been successfully applied to the ecodesign of commercial planes^4^. However, HALE drone mission requirements are very different from commercial planes. In addition to the aforementioned features, flight speed is not a requirement as they fly in closed trajectories. Moreover, a large wing surface area is required in order to gather sufficient solar power. These requirements lead to very different designs, with high aspect ratios. The design of HALE drones has already been studied extensively. Global configuration optimization has been made to an ultralight 3.2 m span solar powered drone using analytical methodology for the conceptual design of such an aircraft^5^. Another related paper focused on the development of a multidisciplinary tool for analysis, design, and optimization of HALE UAVs which act as an “atmospheric satellites” with an extreme aspect ratio concept (500 ft wingspan) using standard approaches, ranging from conceptual design and mission analysis, to preliminary aircraft design methods^6^. In Montagnier et al.^7^ the mass of the flexible wing was minimized using composite materials. The cruise speed versus lift coefficient diagram revealed an optimal solution with a payload of approximately 4 percent of the total mass of 817 kg for a 69 m wing span. From an experimental point of view: scale-sized prototypes have been manufactured, for mechanical testing^8,9^ or aeroelastic studies^10,11^. More recently, a multi-fidelity design vehicle framework has been developed^12^. Botero et al.^13^ applied this framework to a Solar UAV design, highlighting the fact that the energy required for the larger vehicle is approximately twice that of the smaller one (same 10 m wing span but different payload). The vehicles are not optimized for an objective. The optimizer is used to find a feasible vehicle using the mostly energy related constraints. Optimizations seeking higher fidelity were carried out^14^, and a design framework was built^15^. However, all these studies focused solely on mass optimization and did not consider the environmental footprint of the drones. The aim of this work is to close this gap in a global, low-fidelity but fast, environmental impact optimization using open source tools in order to produce reproducible research.

**Figure 1:**
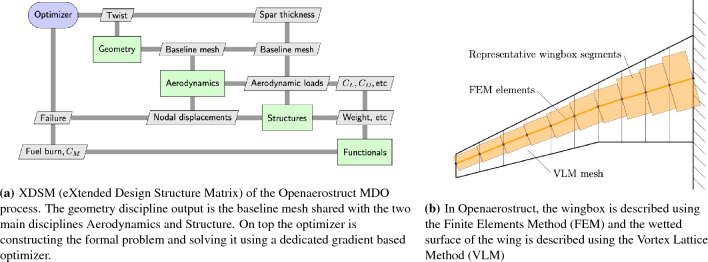
An overview of MDO applied to an aerostructural wing design problem^16,17^.

This work is therefore based on OpenAeroStruct (OAS), a global low-fidelity aerostructural optimization framework^16,17^. This framework is itself based on OpenMDAO^3,18^. The Fig. 1 presents the MDA/MDO process of a typical coupled aerodynamic and structural design problem (OAS), while the next chapter highlights the main differences with the original OAS tool and thus guide the reader to understand the HALE design process starting from a standard aircraft design process.

For a conventional aircraft, most of the $CO_2$ emissions come from the fuel burned. For a solar powered HALE drone, however, no fuel is burned and most of the $CO_2$ emissions come from the materials utilized and the manufacturing of the drone. If the structural material used is fixed, optimizing the environmental impact of the HALE drone is equivalent to optimizing the mass of the drone. Therefore, we decided to include the choice of structural material in the optimization. A recurring problem in optimal material selection is that the material handled as a discrete variable. Another problem is that optimal properties are usually antagonistic. For example a material with a higher Young’s modulus will also have a higher density.

In order to address these difficulties, Ashby indexes can be used^19^. These allow materials to be ordered according to a unique index. However, this method only works when choosing a material for a given simple part under a given loading. This method has been extended for simultaneous material selection and geometry design^20^ and more recently solved using Variational Autoencoder^21^. A new approach has also been developed by the authors to solve material and process selection and thus link ecodesign and topology optimization^22,23^. This method, however, is limited to a structure and cannot take into account the impact of this structure on a bigger system, along with the resulting feedback loops. Making the material variable continuous is a suitable solution for the gradient-based algorithm used in this paper and was first proposed to find the optimal material^24^ and developed in the form of an interpolation in topology optimization^25^. However, the use of this approach in a multidisciplinary design optimization framework is unique in the state of the art and thus constitutes a major contribution of this paper.

The paper is organized as follows. We present in section “HALE drone MDO formulation” the HALE drone model and the optimization framework. Then, we introduce in section “Results and discussion on CO2 footprint minimization” the numerical results and also give some hints on how to interpret them. Finally, we give some concluding remarks in section “Conclusion”.

## HALE drone MDO formulation

### OpenAeroStruct to Eco-HALE

We choose to derive our work from the OpenAeroStruct package with the wingbox full design (^17^). OpenAeroStruct is mainly utilized to study commercial aircraft which we modified to model a HALE drone. To validate our code, we used the single-boom HALE drone test case (data from FBhale publication^14^).

First, as there is no need for a 2.5 G maneuver on a HALE drone, we changed the design points used. We used one cruise flight design point where the lift and power constraints must be satisfied, and one gust design point where the structural constraints must be satisfied.

The OpenAeroStruct constraint on performance, based on the Bréguet equation^26^ cannot be used for a solar-powered HALE aircraft, as the total weight of the aircraft does not change during flight and the range is not limited. The Bréguet range is a simple yet mathematically sound relationship between the main physics (aerodynamic, structural, and propulsion) of the aircraft. Instead, it is a power equilibrium that gives a performance constraint. Actually, the power used by the propulsion and the payload must be produced by the solar panels or stored in the batteries. The power needed for propulsion $P_{prop}$ is derived from the 1D equilibrium between thrust (*T*) and drag (*D*). Thrust and drag are expressed in Eqs. (1) and (2), where *W* is the total weight of the drone, $C_d$ is its drag coefficient, $C_l$ is its lift coefficient, and *v* its speed.

1 $$\begin{aligned} D= & {} W \cdot \frac{C_d}{C_l} \end{aligned}$$

2 $$\begin{aligned} P_{prop}= & {} T \cdot v \end{aligned}$$

These give a link between the power needed for propulsion and the total weight of the drone (Eq. 3).

3 $$\begin{aligned} P_{prop} = W \cdot v \cdot \frac{C_d}{C_l} \end{aligned}$$

The relationship we actually use is Eq. (4), in order to account for the propulsion efficiency $\eta$ and the power needed by the payload and the avionics $P_{payload}$.

4 $$\begin{aligned} P_{needed} = \frac{W \cdot v \cdot C_d}{C_l \cdot \eta } + P_{payload} \end{aligned}$$

During the day, power is supplied by the solar cells. Therefore a minimum wing surface area ($S_{wing}$) is needed in order to place a sufficient number of solar cells. This is expressed in Ineq. (5).

5 $$\begin{aligned} S_{wing} > \frac{P_{needed}}{A_{PV}} \end{aligned}$$

**Figure 2:**
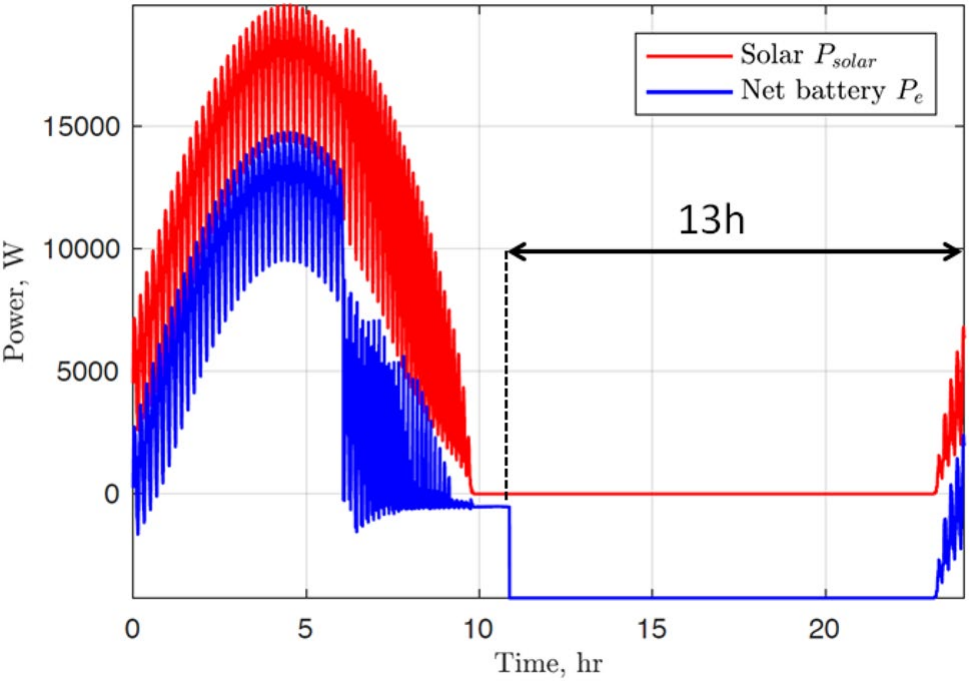
Solar and battery power for FBhale (^14^). The nighttime we considered starts at the end of the gliding phase.

$A_{PV}$ is the power produced per unit of area of solar cells. Ineq. (5) is the constraint on performance we use. $A_{PV}$ is chosen carefully in order to be in the same case as FBhale (^14^). Figure 2 comes from^14^, it was used to extract the mean solar power harvested during the 24h power equilibrium considered. This mean power was divided by the area of solar cells in^14^, and leads to $A_{PV}=54W/m^2$.

**Figure 3:**
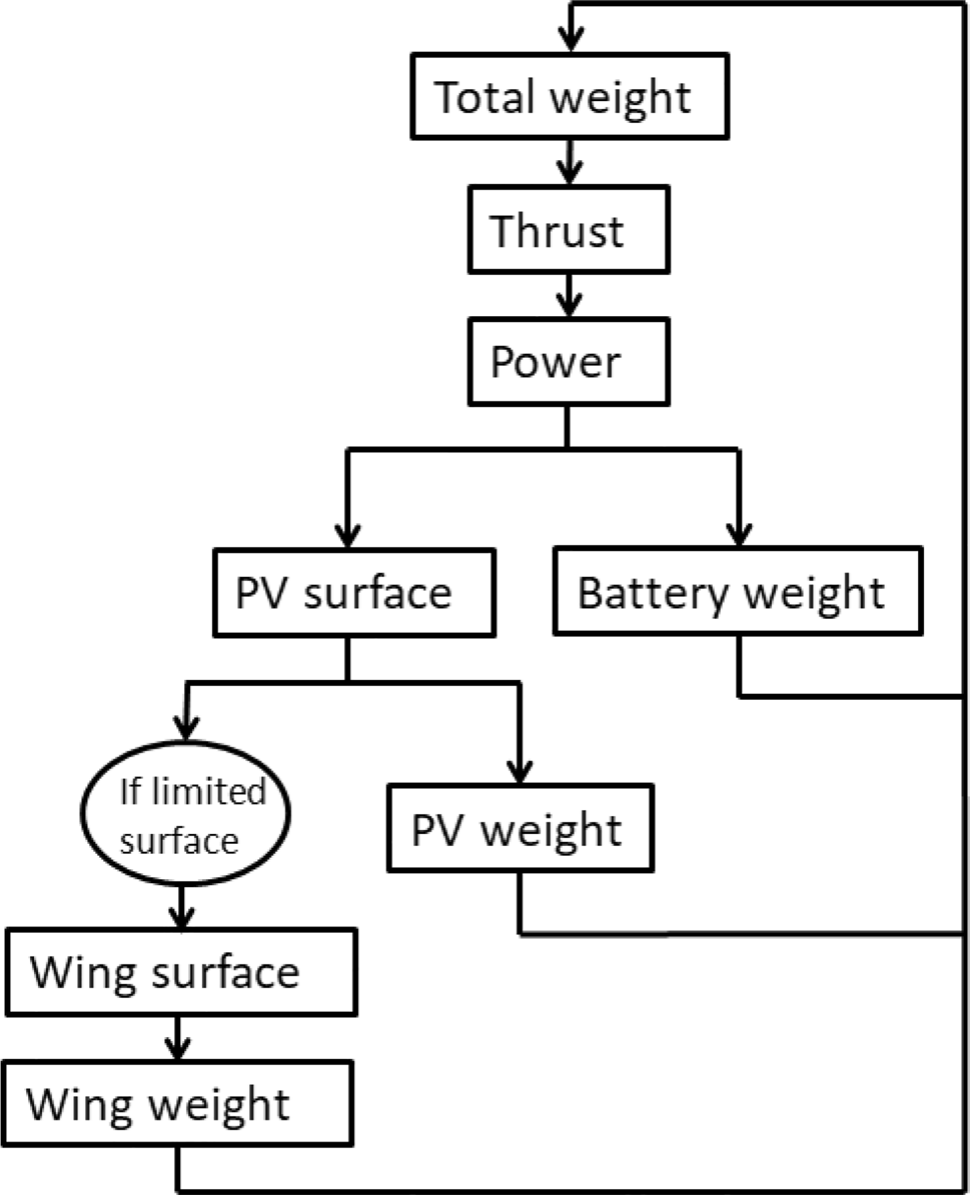
Power and weight coupling: if the total weight changes, the power needed to thrust that weight changes; if the needed power changes, the battery weight, the photo-voltaic cells (PV) weight and possibly the wing weight, change, resulting in a total weight change. This coupling is solved by the same Gauss-Seidel solver as the aerostructural coupling.

The computed surface area of solar cells necessary to produce the needed power is taken into account in the total weight computation, meaning that weight and power are coupled, as can be seen in Fig. 3.

The mass of the solar cells added to the total mass is computed using a surface density from^14^.

The mass of the batteries is also taken into account. In order to compute this mass, an assumption that the batteries power the drone during a 13 h night period ($t_{night}$) is made. This assumption is also based on Fig. 2, and enables a great simplification of the problem by not modeling the flight and gliding phases. This time multiplied by the power needed gives the size of the battery in kWh, which in turn gives the mass of the batteries ($M_{bat}$) when divided by the energy density ($d_{bat}$ in kWh/kg). The energy density was taken equal to that of FBhale. The mass of the batteries is therefore computed with Eq. (6). Taking the battery mass into account adds to the weight and power coupling, as on Fig. 3.

6 $$\begin{aligned} M_{bat}=\frac{P_{needed} \cdot t_{night}}{d_{bat}} \end{aligned}$$

These battery and solar panel masses are distributed along each of the beam elements of the wing, as was done in OpenAeroStruct for the fuel mass. This is closer to reality than a concentrated mass, and relieves the wing, allowing thinner structural skins. Other masses are also taken into account but not distributed, such as the avionics, payload, and the propulsion system mass considered as fixed during optimization.

The propulsion mass ($M_{prop}$) models both the mass of the engine and the propeller. It is obtained as the product of $P_{prop}$ (Eq. 3) and the propulsion density ($d_{prop}$ in kg/W), as in Eq. (7).

7 $$\begin{aligned} M_{prop}=P_{prop} \cdot d_{prop} \end{aligned}$$

In order for the data to be as close as possible to FBhale to validate our framework, without modelling the propeller, this propulsion density is estimated based on^14^. This estimate is obtained by dividing the propulsion mass (extracted from Supplementary materials) by the power used for propulsion (extracted from Fig. 2). These motors and propellers are added as point masses onto the wing structure. As in Eq. (8), the mass of each motor ($M_{mot}$) is obtained by dividing the propulsion mass by the number of motors ($n_{mot}$).

8 $$\begin{aligned} M_{mot}=\frac{M_{prop}}{n_{mot}} \end{aligned}$$

The power system (solar panels, maximum power point tracker and batteries) is sized for a worst case corresponding to the winter solstice where the least solar power is available. By choosing a more favorable launch day, much more power is available during climb. Therefore, the climb phase does not influence the sizing of the power system. As a result, computing the propulsion mass as in Eq. (7) also avoids the need to model the climb phase of the drone. This phase is taken into account through the propulsion mass data from^14^. A shear gust wall is added to the framework, in order to compute the loads used for the sizing of the wings. It has the same magnitude as in^14^. Our model only takes into account the weight of the wing, batteries, solar panels, propulsion, maximum power point tracker (MPPT), avionics (only the fixed mass) and payload mass. In order to account for the extra mass of the harness, the landing gear, the interfaces, the horizontal and vertical tail, the boom and the pod, the total weight of the drone is increased by 10%. This value comes from Supplementary materials (^14^).

**Figure 4:**
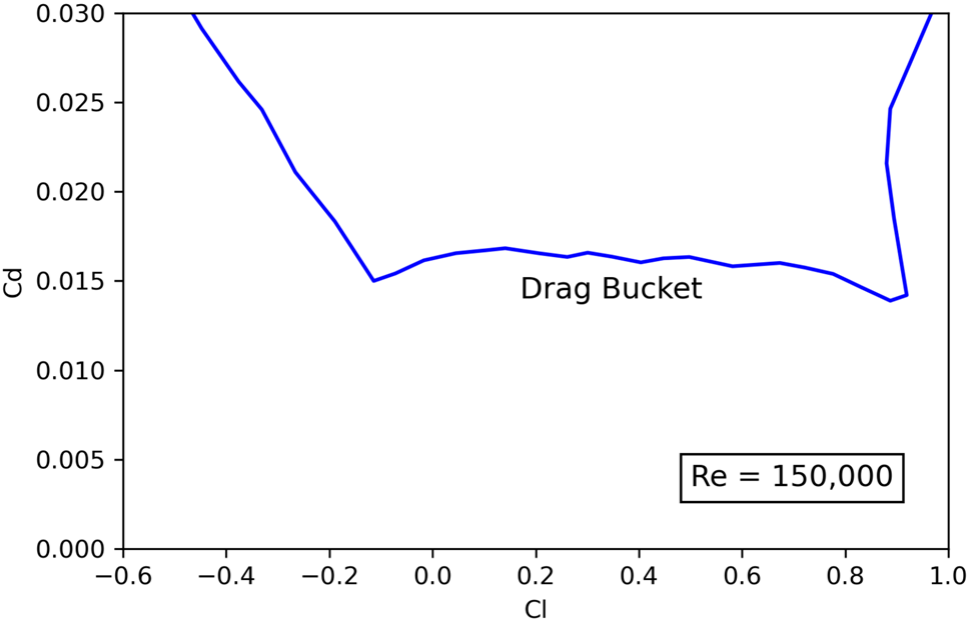
Polar curve for NACA 63412 airfoil.

Finally, the operational conditions of the HALE, such as the high altitude and the low speed, imply a low Reynolds number that lies in the range of 150,000–200,000. For this reason, high values of lift coefficient ($C_l$) are needed and a low-drag configuration is mandatory in order to minimize the power needed for propulsion (Eq. 3). Thus, as proposed in^27^, profile NACA 63412 is selected, which is a laminar airfoil with a high maximum lift coefficient ($Cl_{max}$), a low value of the moment coefficient ($C_m$) and a “drag bucket” covering the desired $C_l$ range (see Fig. 4).

### MDA framework summary

The multidisciplinary design analysis (MDA) framework is summarized in Table 1. The method modeling each discipline is shown in this table, along with the implementation used and a reference.

**Table 1:** Summary of multidisciplinary design analysis framework.

Discipline	Method	Implementation	References
Aerodynamics	VLM	OAS	^28^
Structure	Wingbox beams	OAS	^17^
Energy	Simple in-house method	Section “OpenAeroStruct to Eco-HALE”	Data from^14^
Environmental	Proportional to mass	Section “MDO framework summary”	Data from^29,30^

**Figure 5:**
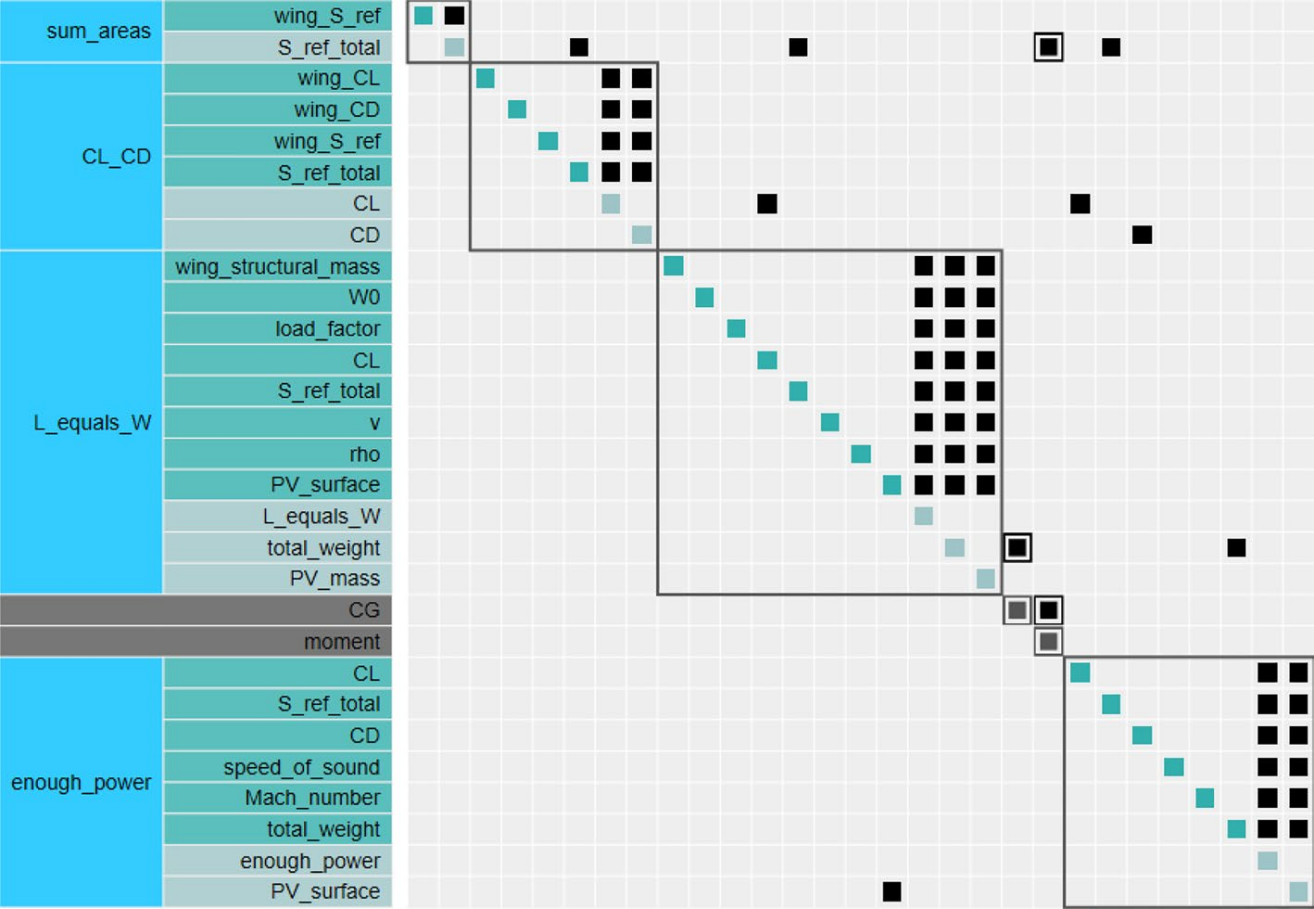
Component layout of the performance group adapted from OpenAeroStruct^16,17^. The coupling between power and weight is visible as a “PV_surface” variable from which the weight of the batteries and solar panels are derived.

MDA converges using the Gauss-Seidel method (also known as fixed-point iterations). Each discipline analysis computes its own set of coupling variables that is passed to other disciplines (analysis). At the end of the MDA process, each discipline returns the final set of coupling variables computed at convergence. Additionally, the chosen optimizer will solve the constrained optimization problem with all the interactions shown in Figure 5.

The energy and environmental models are described in other sections of this paper, listed in Table 1. We briefly present hereafter the aerodynamics and structural models used. These are kept unchanged from the OpenAeroStruct^16^ implementation, apart from the changes to the structure model described in section “MDO framework summary”.

The aerodynamics model is the implementation of the VLM model^28^ by OpenAeroStruct^16^. Each panel of the lifting surface is modeled as a horseshoe vortex. This horseshoe vortex is made of three vortex segments : one span-wise bound vortex located at $\frac{1}{4}\textrm{th}$ of the panel’s chord-wise length and two semi-infinite chord-wise vortices extending towards infinity in the free-stream direction. The total lifting surface is modeled as the superimposition of all these horseshoe vortices. Flow tangency conditions are imposed preventing normal flow through the panel. The aerodynamic forces on each panel are derived from the circulation strength of the vortices. Finally, lift and drag are derived from these forces. Skin friction drag is also estimated. The reader is referred to^16^ for further details concerning this implementation.

The structure model is the beam FEM with wing-box cross-sections implemented in OAS (OpenAeroStruct^17^). The span-wise discretization of this model (7 elements) is the same as for the VLM mesh. Each span-wise section of wing is modeled as a beam element. The cross section of this beam element is a hollow rectangle modeling the upper and lower skins of the wing and the two spars. This model is used to compute the worst case von Mises stress values that are used as a mechanical failure constraint. The reader is referred to^17^ for more detail on this implementation.

### MDO framework summary

#### Adding more physical constraints in OAS

The aim of this part is to add a more detailed sizing process to the existing OAS code and to describe the objective and constraints of the HALE design problem. One has to notice that a mechanical failure constraint leads to insufficient skin thicknesses. Therefore, a new (linear) buckling constraint is added in the design process. This buckling constraint imposes that the stress in the top skin of each element stays below the buckling critical stress of that panel. In order to have only one constraint, a Kreisselmeier-Steinhauser (KS) aggregation is used (^31,32^), as for the initial mechanical failure constraint. The buckling critical stresses are estimated by the case of a homogeneous rectangular curved plate subject to combined axial compression and shear. Therefore, two different buckling critical stresses are considered: an axial critical stress ($\sigma _c$) and a shear critical stress ($\tau _c$), which can be expressed in the form of Eqs. (9) and (10), respectively, where $k_c$ is the buckling coefficient for axial load, $k_s$ the buckling coefficient for shear, *b* the width of the panel, and *D*, the flexural stiffness. In our approximate buckling case, we take *b* to be the distance between the two spars (set at half the chord) and the values of $k_c$ and $k_s$ can be obtained graphically from the charts presented in^33^, using the geometry of the panel as an input.

9 $$\begin{aligned} \sigma _c= & {} k_c \cdot \frac{\pi ^2 \cdot D}{b^2} \end{aligned}$$

10 $$\begin{aligned} \tau _c= & {} k_s \cdot \frac{\pi ^2 \cdot D}{b^2} \end{aligned}$$

Thus, the buckling constraint is approximated by a parabolic interaction, as in Ineq. (11), proposed in^33^, $R_s$ and $R_c$ being the stress ratios for shear and axial compression, respectively. These stress ratios are defined as the ratio of the stress at buckling under combined loading to the buckling stress under simple loading.

11 $$\begin{aligned} R_s^2 + R_c < 1 \end{aligned}$$

We also add a constraint imposing that the skin thickness cannot be greater than half the wing thickness, in order to prevent the top and bottom skin from intersecting at the wing tips, where the chord is smaller and the thickness-to-chord ratio of the wing is is also small. To simplify the MDO process, the lower bound is fixed at 1 mm for all materials such as homogeneous metals or homogenized composites materials. At the conceptual detailed design level this limitation can be easily treated.

#### New objective function: CO2 footprint

We choose the $CO_2$ emitted by the HALE drone during its life cycle ($CO2_{tot}$) as our objective function. As the drone does not burn any fuel during flight, it is assumed that the $CO_2$ is mainly emitted before the use of the drone and is in particular due to the materials used and their processing. As the aim is to have a simple and fast tool, we consider only the $CO_2$ emitted by the material used for the structure ($CO2_{struct}$), the $CO_2$ emitted by the solar panels ($CO2_{PV}$), and the $CO_2$ emitted by the batteries ($CO2_{bat}$), as these have the most influence on the total emitted $CO_2$. This is shown in Eq. (12).

12 $$\begin{aligned} CO2_{tot} = CO2_{struct} + CO2_{PV} + CO2_{bat} \end{aligned}$$

The $CO_2$ emitted by the structure is computed as the product of the mass of the spars ($M_{spar}$) by the $CO_2$ footprint of the material used for the spars ($CO2_{mat1}$) and the product of the mass of the skins ($M_{skin}$) by the $CO_2$ footprint of the material used for the skins ($CO2_{mat2}$), as shown in Eq. (13). This $CO_2$ footprint of the materials is considered to be equal to a weighted sum of the $CO_2$ footprint of their primary production ($CO2_p$) and the $CO_2$ footprint of their recycling ($CO2_r$), depending on their recycled fraction in current supplies ($\eta _r$), as in Eq. (14).

13 $$\begin{aligned} CO2_{struct}= & {} M_{spar} \cdot CO2_{mat1} + M_{skin} \cdot CO2_{mat2} \end{aligned}$$

14 $$\begin{aligned} CO2_{mat}= & {} \eta _{r} \cdot CO2_{r} + (1-\eta _{r}) \cdot CO2_{p} \end{aligned}$$

It can be seen in Eqs. (13) and (14), that the $CO_2$ footprint of the processing of the materials is not taken into account. We made this choice because $CO_2$ footprint of processing varies a lot depending on the process, and we have no information related to this stage. This choice is acceptable as the footprints of the most probable processes are much lower than the footprint of the material primary production, and this is especially true for the materials that were found to be optimal.

The $CO_2$ emitted by the solar panels is computed as the product of the power needed on board the HALE ($P_{needed}$ detailed in section “OpenAeroStruct to Eco-HALE”) and the emissions per power ($CO2_{/W}$), as in Eq. (15). The value of $CO2_{/W}$ is taken from^29^.

15 $$\begin{aligned} CO2_{PV} = P_{needed} \cdot CO2_{/W} \end{aligned}$$

The $CO_2$ emitted by the batteries is computed as the product of the size of the battery ($P_{needed} \cdot t_{night}$ as in section “OpenAeroStruct to Eco-HALE”) and the emissions per Wh ($CO2_{/Wh}$), as in Eq. (16). The batteries are assumed to be similar to electric car Li-ion batteries, and the value of the emissions per Wh is taken equal to the mean of the three batteries studied in^30^.

16 $$\begin{aligned} CO2_{bat}=P_{needed} \cdot t_{night} \cdot CO2_{/Wh} \end{aligned}$$

#### Design variables

We used eight geometric design variables and a material design variable which we discuss in detail in section “MDO framework summary”. B-splines parameterized using four control points each were adopted in order to vary wing geometric design variables along the semi-span. The only angle design variable we used was the vector of control points for the twist, because it also enabled us to control the angle of attack. Therefore, our variable twist represents the sum of geometric twist and angle of attack at cruise conditions.

We kept the wingbox design variables of OpenAeroStruct, which are the control points for the skin thickness and the spar thickness. These are mainly determined by the buckling constraint and the mechanical failure constraint, respectively. We used the same thickness distribution for both the upper and lower skins, and the same thickness distribution for both the forward and rear spars.

In order to satisfy both the power constraint and the weight-equals-lift constraint, we kept the control points for wing geometry as thickness-to-chord ratio, span, chord and taper ratio as design variables. These variables can generate the particular geometries of HALE drones with very high aspect ratios.

Additionally, the motor spanwise location was added as a new design variable. Since we are considering a symmetrical twin-motor HALE, this new design variable is defined as the ratio of the distance between the plane of symmetry and the motor to the semi-span of the wing.

Some limitations must be imposed to the design space in order to compensate for some missing physics in the model. Wings cannot be too tapered because tip stall can happen otherwise. As stall is not modelled in our framework, we choose a lower limit of 0.3 for the taper ratio design variable. For the same reason, a lower and upper twist limits of − 15 and 15 degrees are considered. In this way, the critical or stalling angle of attack is taken into account. Furthermore, wings cannot be too narrow because 1−cosine gusts limit their aspect ratio. These gusts are not modelled, but the rule exists in the FAA (Federal Aviation Administration) regulation, so we also added a lower limit of 1.4 m for the root chord.

#### Material selection

The battery and solar panel types are fixed in our case. Therefore, minimizing the $CO_2$ emissions due to the solar panels and batteries, consequently, minimizes the power needed, which means minimizing the mass of the drone to be thrusted. Similarly, minimizing the $CO_2$ emissions due to the structural materials means minimizing the mass of materials used, if the choice of materials is not a variable. Therefore, if the structural materials were fixed, optimizing the $CO_2$ impact of the drone would be equivalent to optimizing the mass of the drone. Therefore, we chose to add the choice of the structural materials as a design variable. This new additional design variable takes the form of a vector with two components: the density of the material used for the spars and the density of the material used for the skins. Distinguishing these two structural elements adds new interesting degrees of freedom to our problem.

One of the main simplifications of our approach, is the material selection through their density. For the sake of simplicity this choice is also linked to the MDO process that needs to select a unique material at the end. The material data we need (Young’s modulus, shear modulus, yield strength, and $CO_2$ emissions) is therefore accessed as a function of the density design variable. This limitation was solved in one of our recent publications^22^.

**Figure 6:**
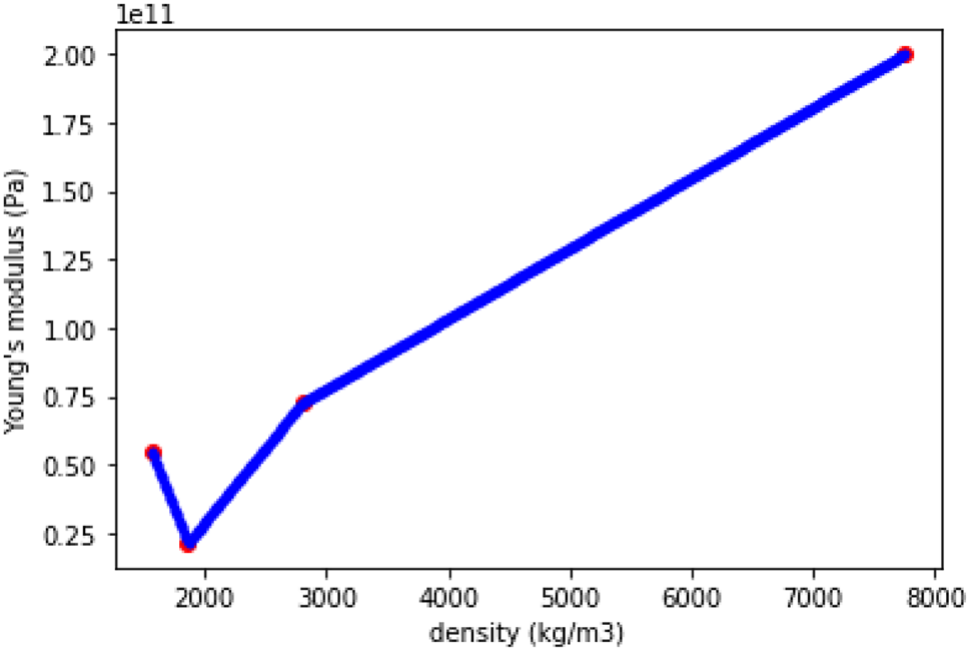
Young’s modulus example of linear interpolation of materials.

In order to have only continuous variables, we decide to make the density design variable continuous. This is achieved by linearly interpolating each material property in the space between real materials. The resulting interpolation is shown on Fig. 6 for two isotropic homogenized materials: Carbon Fiber Reinforced Composite (CFRP), Glass Fiber Reinforced Composite (GFRP) and two homogeneous metals: aluminium and steel. This is one of the limits of our approach, always linked to the simplification of the MDO process. Of course, this will lead to sub-optimal composite materials compared to more detailed structural sizing process.

However, with this interpolation, it is possible to find an optimum in an interpolated material corresponding to no real material. In order to eliminate this possibility, the interpolation is penalized by the use of a power term in the interpolation, as in^25^. An example of this interpolation for Young’s modulus is given in Eq. (17), where *E* is the interpolated Young’s modulus at density $\rho$, $\rho _i$ and $\rho _{i+1}$ are the densities of the real materials framing the one being interpolated, $E_i$ and $E_{i+1}$ are the respective Young’s modulus of these real materials, and *p* is the power used for penalization.

17 $$\begin{aligned} E(\rho )=A \cdot \rho ^p+B \end{aligned}$$

with $$A=\frac{E_{i+1}-E_i}{\rho _{i+1}^p-\rho _i^p}$$ and $$B=E_i-A \cdot \rho _i^p$$

**Figure 7:**
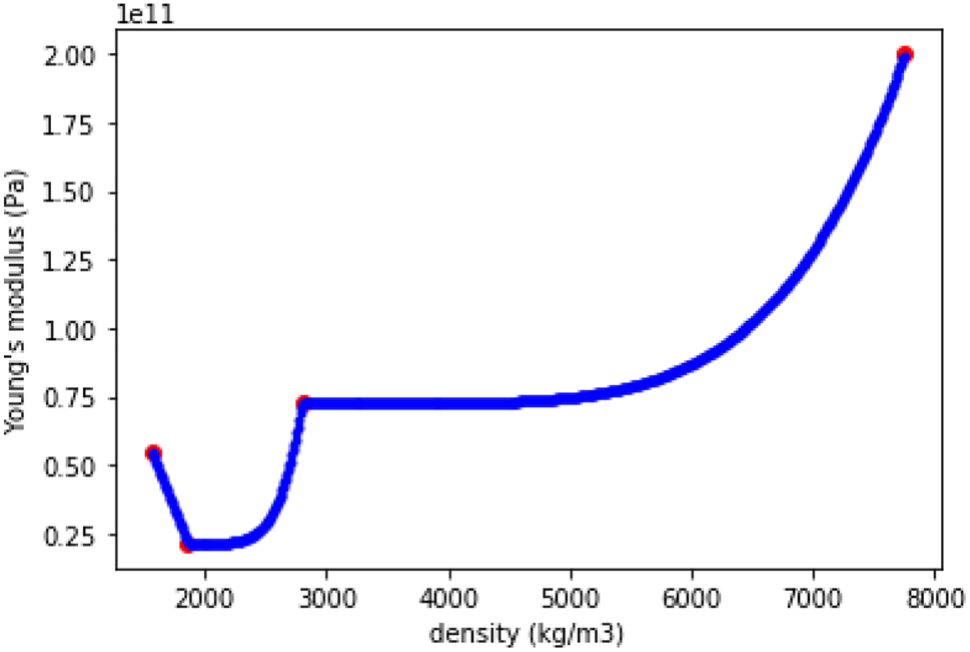
Young’s modulus example of penalized interpolation of materials.

The resulting interpolation is shown on Fig. 7 for $p=5$, and for the same real materials as on Fig. 6.

To the left of a real material, a small decrease in density brings for example a great decrease in Young’s modulus. Therefore, the slight advantage due to a slightly lower density is compensated by the Young’s modulus evolution. To the right of a real material, even a big increase in density brings a very small increase in Young’s modulus. Therefore, the slight advantage due to a slightly higher Young’s modulus is compensated by the higher density. As a result, choosing a material that is not real (non-physical) is not beneficial, and the final optimum will be a real material.

**Figure 8:**
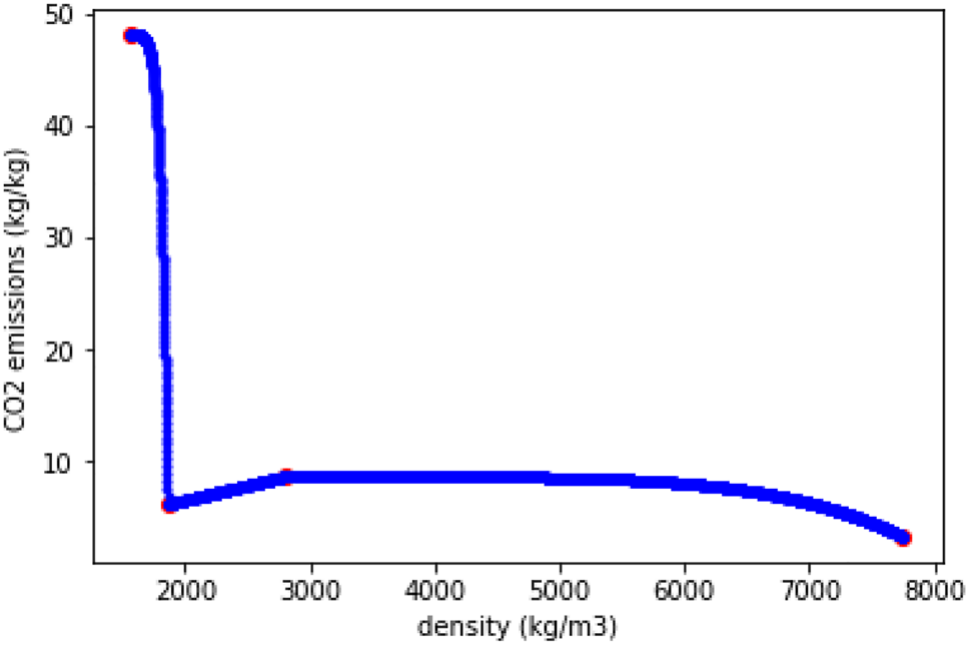
$CO_2$ emissions example of penalized interpolation.

In order to not advantage these non-physical materials, the penalization isn’t used in the case where a lower density leads to a better property. This appears for example in the left-hand part of Fig. 7, between CFRP and GFRP, where a decrease in density leads to an increase in Young’s modulus. For material properties for which a smaller value is better, such as $CO_2$ emissions, the penalization is inverted, as in Fig. 8.

This penalization must be applied to the interpolation only at the end of the optimization. In fact, as we use a gradient optimization, once the penalization is given to the interpolation, the solution is stuck in the local minimum corresponding to the closest material. The optimal material being an easily identified angular point (for example in Fig. 8), it is often also the optimum found even without any penalization in the interpolation. However, adding the penalization is more robust. Table 2 summarizes the MDO framework.

**Table 2:** Summary of multidisciplinary design optimization framework.

Objective function	Dimension	Bounds
$CO2_{tot}$	$\mathbb {R}$	
Design variables		
Density	$\mathbb {R}^2$	[400, 8000] kg/m$^3$
Twist control points	$\mathbb {R}^4$	[− 15, 15] deg
Skin thickness ($t_{skin}$) control points	$\mathbb {R}^4$	[0.001, 0.1] m
Spar thickness control points	$\mathbb {R}^4$	[0.001, 0.1] m
Thickness-to-chord ratio control points	$\mathbb {R}^4$	[0.01, 0.4]
Span	$\mathbb {R}$	[1, 1000] m
Root chord	$\mathbb {R}$	[1.4, 500] m
Taper ratio	$\mathbb {R}$	[0.3, 0.99]
Motor location over semi-span ratio	$\mathbb {R}$	[0, 1]
Constraints		
Mechanical failure $\sigma < \sigma _{max}$	$\mathbb {R}^7$	
Buckling $R_s^2 + R_c < 1$	$\mathbb {R}^7$	
Skin thickness $2 t_{skin} < t_{wing}$	$\mathbb {R}^4$	
Power equilibrium $P_{needed} / A_{PV} < S_{wing}$	$\mathbb {R}$	

It is important to highlight that the SLSQP (Sequential Least Squares Programming) optimization algorithm is used in this work. The chosen optimizer options (stopping criteria) are $10^{-3}$ for the convergence accuracy and 250 for the maximum number of iterations. We checked the analytical derivatives using the complex step method^34^.

## Results and discussion on $CO_2$ footprint minimization

Once the framework was validated (see Supplementary materials), an optimization was run. It should be recalled that our goal was to optimize the $CO_2$ footprint of the HALE, therefore, the objective function is now this total $CO_2$ emitted. Nevertheless, gradient based solvers converge to a locally optimal point, so the search for a global optimum depends heavily on the starting point of the optimization. For this reason, the multi-start strategy described in Table 3 was used.

**Table 3:** Design variable starting values for multi-start strategy.

Design variable	Lowest starting value	Highest starting value	Number of starting values	Unit
Density	[500 500]	[600 600]	2	kg/m$^3$
Twist control points	[10 15 15 15]	–	1	deg
Skin thickness control points	0.002$\cdot$[0.5 1 1.5 2]	0.004$\cdot$[0.5 1 1.5 2]	3	m
Spar thickness control points	0.001$\cdot$[1 1 1 1]	0.003$\cdot$[1 1 1 1]	3	m
Thickness-to-chord ratio control points	0.05$\cdot$[0.75 1 1 1.25]	0.17$\cdot$[0.75 1 1 1.25]	3	–
Span	25	100	4	m
Root chord	1.5	–	1	m
Taper ratio	0.3	–	1	–
Motor location over semi-span ratio	0.3	–	1	–

Every combination of values is used, meaning that a total of $2 \cdot 3 \cdot 3 \cdot 3 \cdot 4 = 216$ optimizations are run. For each variable, the values are regularly spread between the highest and lowest. For example, for the skin thickness control points, the three values given at the start to that variable are [0.001 0.002 0.003 0.004], [0.0015 0.003 0.0045 0.006], and [0.002 0.004 0.006 0.008].

The optimization was run with seven homogeneous materials integrated into the material design variable. Their properties are shown in Table 4.

**Table 4:** Material properties.

Property	Material 1	Material 2	Material 3	CFRP	GFRP	Aluminum	Steel	Unit
Density	504.5	529	560.5	1565	1860	2800	7750	kg/m$^3$
$CO_2$ emissions	44.9	42.8	40.3	48.1	6.18	8.66	3.28	kg$_{CO_2}$/kg
Young’s modulus	42.5	42.5	42.5	54.9	21.4	72.5	200	GPa
Shear modulus	16.3	16.3	16.3	21	8.14	27	78.5	GPa
Failure strength	587	237	587	670	255	445	562	MPa
Buckling index	0.1539	0.1543	0.1544	0.05049	0.24153	0.1720	0.2302	N$^{\frac{1}{3}} \cdot$m$^{\frac{7}{3}}$/kg$_{CO2}$
Strength index	25885	10484	25959	8901	22184	18331	22119	N$\cdot$m$\cdot$kg$_{CO2}$

Those named material 1, 2 and 3 are roughly homogenized sandwiched panels. Their skins are of the same carbon fibre reinforced polymer (CFRP) as the CFRP material in Table 4. The expanded polystyrene (PS) foam core of material 1 is replaced by a balsa core in material 2 and a cork core in material 3. Those cores are heavier than the one of material 1 but emit less $CO_2$ per kilogram of material.

Two material indices are computed according to the method of^19^. Both indices correspond to minimizing the $CO_2$ emitted by the structure $CO2_{struct}$, (defined in Eq. 13), for a flat plate loaded with in-plane compression. The buckling index is useful in the case where the buckling constraint described in section “MDO framework summary” is active. In this case, the index to be maximized can simply be established in 18 as:

18 $$\begin{aligned} E^{1/3}/\rho /CO2_{mat} \end{aligned}$$

where E is the Young’s modulus of the material, $\rho$ its density, and $CO2_{mat}$, the $CO_2$ footprint of the material (defined in Eq. 14). The strength index is useful in the case where the mechanical failure constraint is active. In this case, the index to be maximized is defined in (19):

19 $$\begin{aligned} \sigma _{f}/\rho /CO2_{mat} \end{aligned}$$

where $\sigma _f$ is the failure strength (yield strength or tensile strength depending on the material). These material indices appear in Table 4.

**Figure 9:**
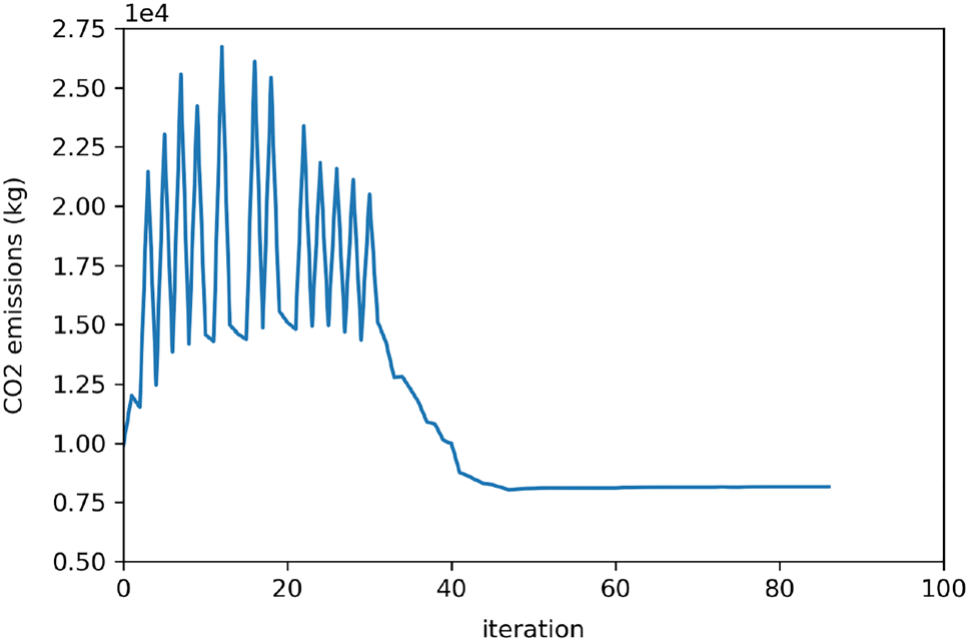
Objective function: total $CO_2$ emitted by the drone.

**Figure 10:**
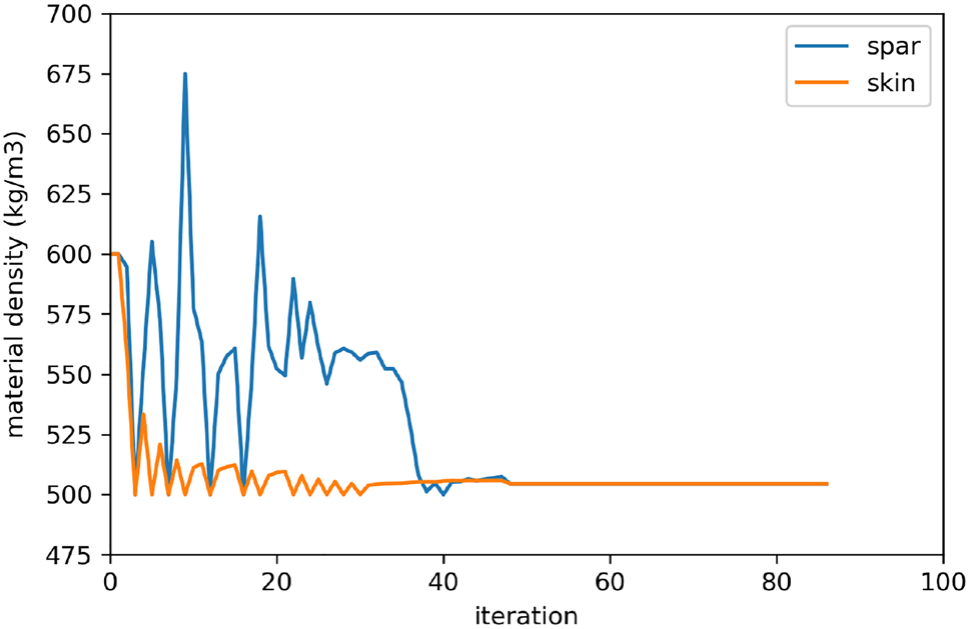
Material density convergence graph.

The $CO_2$ emissions and density convergence graphs obtained during optimization are shown on Figs. 9 and 10, respectively.

The convergence graphs demonstrate that the lightest material is selected for both spars and skins.

**Figure 11:**
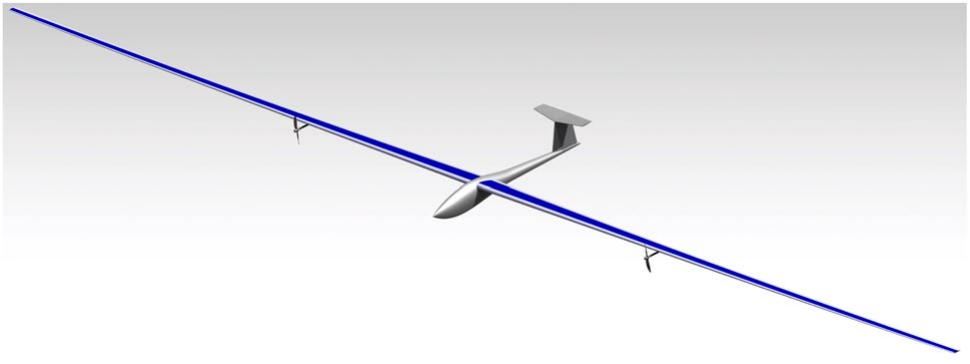
Rendering of the CAD model (using CATIA) for the optimal HALE wing.

Additionally, the CAD model of the resulting HALE is shown in Fig. 11 (with generic boom and tail shapes since only the wing structure is optimized). The optimization converges towards Material 1 for both spars and skins. This material is the one with the lowest density among those accessible through the material design variable. However, it is not the one with the best $CO_2$ emission indices. Indeed, the density indices for Materials 2 and 3 are both higher than those of Material 1. The optimization was re-run with fixed-material (1, 2 or 3) and confirmed that Material 1 performs better. This material is also the optimal material in case the total weight of the drone is the objective function. This optimization clearly confirms that in our specific case of an electric HALE drone, minimizing the mass is equivalent to minimizing the CO2 footprint. As expected, a slight increase in the total weight of the drone leads to an increase in the weight of the battery and the solar panel in order to propel a heavier drone, and also to an increase in the weight of the wing structure that induces a more important lift to compensate. These weight increases contribute to a further increase in the overall weight of the drone. This is called the “snowball” effect.

This result can be explained by the fact that the $CO_2$ emissions taken into account in the objective function are not only due to the structure (Eq. 12) explains this result. Although the structure composed of Material 3 emits less $CO_2$ than the one with Material 1, it is also heavier. The structure being heavier with Material 3, it needs more power to be thrusted, and thus, more $CO_2$ is emitted by the batteries and solar panels. This compensates the lower emission due to the structure. As a result, material indices as in^19^ can’t be used in this case. A direct ratio between the weight of the structure and the $CO_2$ emitted by the batteries and solar panels would be needed in order to have a truly useful material index. However, this ratio is not accessible as it evolves during optimization. Therefore, a method such as the gradient optimization proposed in this work is necessary to choose the best material, if all material candidates can’t be tested individually.

**Figure 12:**
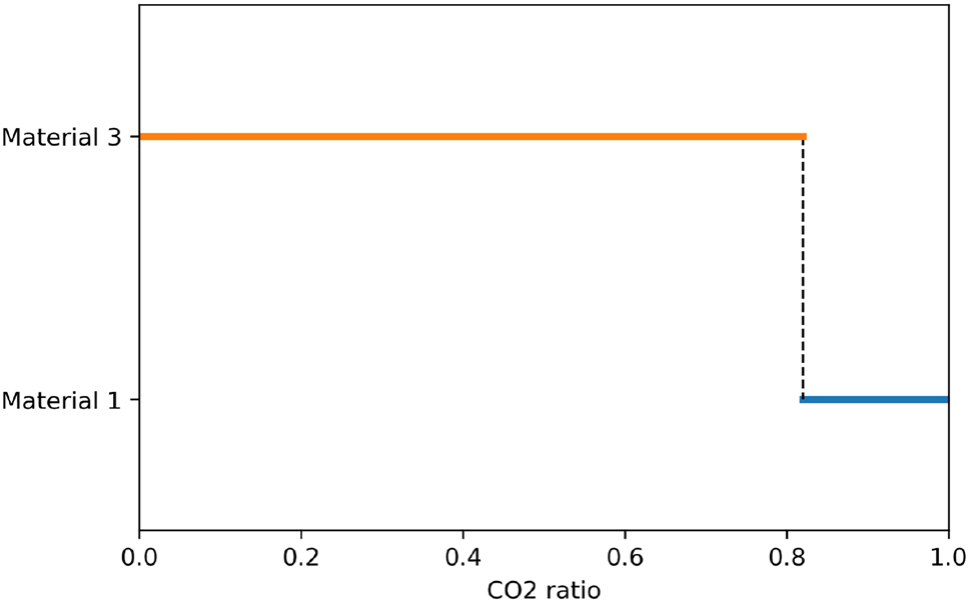
Optimal material depending on changes to the $CO_2$ emissions of Material 3. $CO_2$ ratio is the ratio of the $CO_2$ emissions of Material 3 to the $CO_2$ emissions of Material 1.

An informal test was carried out to compare the relative influence of the density and the $CO_2$ emissions of a material on its being optimal. For this test, the $CO_2$ emissions of Material 3 are lowered until this material becomes optimal. In this test, Material 1 and Material 3 differ only by their densities and $CO_2$ emissions. The density of Material 3 is 11% higher than that of Material 1. The results are presented in Fig. 12. We can note that the $CO_2$ emissions of Material 3 must be approximately 20% lower than those of material 1, in order to compensate for its 11% higher density. It is reminded that Material 3 is the eco-material surrogate for Material 1, the expanded PS foam core of Material 1 being replaced by a cork core in Material 3. Therefore, in this case, in order to lower the total $CO_2$ emissions of the drone, an eco-material surrogate must also be almost as good as the initial material in terms of weight.

The code (see section Data Availability) is not optimized in terms of computational time. This will be addressed in future work. However, the speed of the optimization method presented in this article does not depend on the number of materials in the material catalogue. It is therefore particularly well-suited to large catalogs in comparison to a brute force method consisting in optimizing the structure for every material in the catalog. We also wish to emphasize that the results of our low fidelity tools are in very good agreement with the medium fidelity tools used for the design of FBhale^14^ as demonstrated in the mass minimization problem applied to FBhale (see Supplementary materials).

## Conclusion

The authors have developed a low fidelity MDO framework for preliminary design of solar powered HALE. The code is opensource and available for the community. Material choice was successfully integrated in a continuous multidisciplinary design optimization regarding $CO_2$ emissions. The simple HALE model developed showed acceptable agreement with more complex models such as FBhale, which was designed using much more sophisticated and costly tools.

Our modified version of OpenAeroStruct includes certain improvements with respect to the original version of the tool. For instance, it is adapted to HALE drones and contains more physics: material choice from among a discrete catalogue, batteries, solar panels, buckling constraint, etc.

A key result is that an optimal material in terms of drone total weight is also optimal in terms of drone total $CO_2$ emissions, even for an electrical drone. In order to be competitive in terms of total $CO_2$ emissions, an eco-material substitute must be almost as good as the initial in terms of drone total weight. This is due to a “snowball” effect on weight. One solution for tackling this problem is to generate this eco-material substitutes by creating digital materials based on unit cell optimization and apply this multiscale topology scheme to ribs and spars^23,35^.

It is true that HALE are highly flexible aerostructures. Thus more extensive sizing such as aeroelastic analysis and gust response should be conducted in a near future.

Finally, a good feature that could be addressed in the future would be a multi-objective optimization between $CO_2$ footprint and cost.

## Supplementary Information

Supplementary Information.

## Data Availability

The optimization presented in the results can be obtained by following the instruction of the file howToStart at: https://github.com/mid2SUPAERO/ecoHALE/tree/downloadEcohale. The datasets used and/or analyzed during the current study are available from the corresponding author on reasonable request.
