# Supplementary material for: CO2 footprint minimization of solar-powered HALE using MDO and eco-material selection
Source: Sci Rep. 2023 Jul 25;13:11994. doi: 10.1038/s41598-023-39221-3 (PMC10368653; doi:10.1038/s41598-023-39221-3)
Supplement: Supplementary file 1 — Supplementary Information. [file 41598_2023_39221_MOESM1_ESM.pdf]

Supplementary materials

A1: Mass minimization problem (validation with FBhale)

In order to validate our fast and low fidelity framework, we compared it to[?] on a similar run. For the comparison to be significant, we used the same data as[?] whenever possible. For this validation, we also changed our objective function in order to conduct a total mass optimization as in[?], instead of a CO_2 optimization. Finally, we fixed the material design variable to a material similar to that of[?].

We obtain the convergence graphs of Fig. 1, and the final design variable values of Tab. 1.

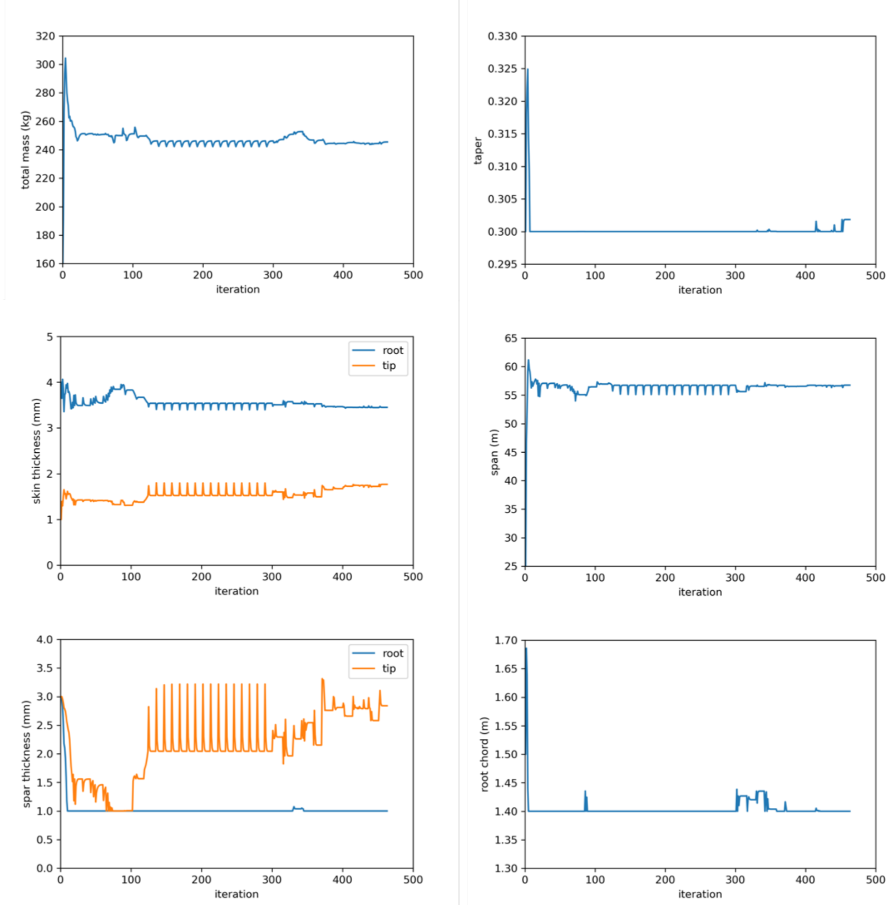

Supplementary Figure 1. Convergence graphs of some design variables for validation case. All final values are summarized in Tab. 1. Most of the design variables tend to a stable final value except for the spar thickness at the wing tip which fluctuates.

We added other variables to this table and the values for[?] as well. Our optimization results are of the same order as those of[?]. In particular we can see in Supplementary Fig. 2 that the mass is similarly distributed between the different parts of the drone.

We can see that the share of the weight due to propulsion is nearly the same in both studies. This is expected as we derived our propulsion data from[?] in order to achieve this (see section **??**). However, the proportions of the weight due to the batteries, the wing and the solar panels are also similar in both studies. This shows that our modelling is a good approximation of this higher-fidelity modelling.

There are however some differences, in particular regarding the aspect ratio. Those are mainly due to our not taking into account 1-cosine gusts in our framework. Indeed, we only take into account a shear gust wall, whereas[?] also takes into account a 1-cosine gust, which leads to more stress than a gust wall for a large-span wing. This difference over-penalizes large chords and under-penalize long spans, resulting in a higher aspect-ratio. However, the exact shape of the wing was not the main focus of this work, but rather the coupling between structural mass and total drone mass. This coupling is necessary to ascertain the global influence of the choice of structural materials, and is well-captured here. A small difference in data can have a big impact on the final mass of the drone. Indeed, a small increase in the drone's total weight, leads to an increase in battery and solar panel weight in order to thrust the heavier drone, and to an increase in wing structural weight to lift the heavier drone. These weight increases contribute to a further increase in the drone's total weight. This is called the "snowball" effect. For

Supplementary Table 1. Final design variable values for validation case.

Variable	Final values for our case	Final values for FBhale	Unit
Twist control points	[6 12 14 15]	-	deg
Skin thickness control points	[1.8 1.7 2.4 3.5]	-	mm
Spar thickness control points	[2.8 1.1 1.0 1.0]	-	mm
Thickness-to-chord ratio control points	[0.10 0.22 0.23 0.24]	-	-
Span	53.5	45.6	m
Root chord	1.4	-	m
Taper ratio	0.3	-	-
Total mass	245	320	kg
Wing surface	50.3	71.8	m^2
Aspect ratio	57	29	-
C_L at cruise	1.39	1.33	-
$C_L^{3/2}/C_D$ at cruise	50.2	40.1	-
Motor location over semi-span ratio	0.33	0.46	-

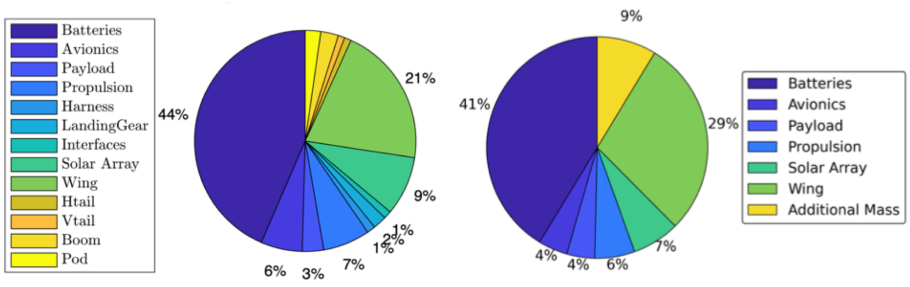

Supplementary Figure 2. Comparison of the mass breakdowns for FBhale's single-boom (left) and our code validation (right). The mass is similarly distributed between the different parts of the drone in both cases, even though FBhale was made using much more complex tools in the design loop.

example, the fixed mass of the drone is only 20.5 kg (payload and avionics), but in order to thrust and lift that mass, the final optimized mass of the drone is more than 10 times that.
